# Could vitamin D concentration be a marker of a long hospital stay in older adults patients?

**DOI:** 10.3389/fnut.2023.1277350

**Published:** 2023-10-20

**Authors:** Justyna Nowak, Marzena Jabczyk, Paweł Jagielski, Bartosz Hudzik, Katarzyna Brukało, Jakub Borszcz, Barbara Zubelewicz-Szkodzińska

**Affiliations:** ^1^Department of Metabolic Disease Prevention, Faculty of Public Health in Bytom, Medical University of Silesia, Bytom, Poland; ^2^Department of Nutrition and Drug Research, Faculty of Health Sciences, Institute of Public Health, Jagiellonian University Medical College, Krakow, Poland; ^3^Third Department of Cardiology, Silesian Center for Heart Disease, Faculty of Medical Sciences in Zabrze, Medical University of Silesia, Zabrze, Poland; ^4^Department of Health Policy, Faculty of Public Health in Bytom, Medical University of Silesia, Bytom, Poland; ^5^Student Scientific Circle Affiliated of Department of Metabolic Disease Prevention, Faculty of Public Health in Bytom, Medical University of Silesia, Bytom, Poland; ^6^Department of Endocrinology, District Hospital, Piekary Śląskie, Poland

**Keywords:** vitamin D, vitamin D deficiency, older adults patients, long hospital stay, biomarker

## Abstract

**Background and aims:**

Vitamin D plays a pleiotropic role in the human body. Some studies have suggested that hypovitaminosis D may serve as a marker of comorbidity severity and length of hospital stay. Hospitalized older adults patients with a higher comorbidity burden tend to have lower vitamin D status, which negatively impacts the length of their hospital stay. Vitamin D deficiency has been identified as a significant risk factor for a prolonged hospital stay. This study aimed to investigate the link between vitamin D status and prolonged hospital stays, focusing on geriatric patients, and to assess the variation in hospitalization duration among geriatric patients with different vitamin D statuses.

**Methods:**

The study sample comprised of 422 patients aged over 60 years admitted to the geriatric department. Blood samples were collected in the morning on the day of admission. According to the diagnostic threshold defining serum 25(OH)D concentration approved for Central Europe, patients were divided into two groups (deficiency group and suboptimal group). Patients were divided into two groups based on hospitalization duration: the first, “shorter hospitalization,” included stays up to 11 days, whereas the second, “longer hospitalization,” encompassed stays of 12 days and above.

**Results:**

In total, 242 Caucasian patients, primarily women (172 women and 70 men), were recruited in the study. Patients with vitamin D deficiency had extended hospital stays compared with those with vitamin D levels below 49.92 nmol/L: 10.0 (8.00–13.00) days vs. 9.00 (8.00–11.00) days, *P* = 0.044. Hospitalization length (in days) had a negative correlation with vitamin D blood status (nmol/L) (*P* = 0.0005; *R* = −0.2243). ROC analysis indicated that patients with vitamin D levels below 31.2 nmol/L had a 47% higher chance of extended hospitalization, whereas those with levels above 31.2 nmol/L had a 77% higher chance of avoiding it. A significant majority of patients with suboptimal 25(OH)D levels experienced shorter hospital stays (≤11 days) than those with vitamin D deficiency (64.6%), *P* = 0.045.

**Conclusion:**

The study findings indicate that lower serum levels of 25(OH)D in hospitalized patients within the geriatric department are linked to extended hospital stays. Vitamin D holds potential as a predictor of hospitalization duration in geriatric patients. Nonetheless, further research is imperative to account for additional factors affecting health status and hospitalization duration in older adults individuals.

## Introduction

Vitamin D plays a crucial role in the muscular, immune, endocrine, and central nervous systems (1) and in calcium and bone homeostasis. Vitamin D may have potential benefits in reducing symptoms of depression (2). Vitamin D supplementation benefits patients with type 2 diabetes (e.g., lower effect on HOMA-IR, HbA1c, and insulin levels), and vitamin D supplementation might be considered as a beneficial dietary component in managing hyperglycemia (3). Vitamin D deficiency may be associated with a higher risk of cardiovascular diseases, some cancers, and Alzheimer's disease. Meta-analysis studies confirm the potential benefits of vitamin D supplementation in reducing SBP (systolic blood pressure) and DBP (diastolic blood pressure) levels (4). Recently, some studies have suggested that hypovitaminosis D may serve as a marker of comorbidity severity and length of hospital stay (LOS) (5, 6). Hospitalized older adults patients with a higher comorbidity burden tend to have lower vitamin D status, which negatively impacts the length of their hospital stay (7). Other studies have reported similar findings, showing inverse linear correlations between serum 25(OH)D concentrations and the length of hospital stay among older adults patients (8, 9). Vitamin D deficiency has been identified as a significant risk factor for an extended hospital stay (10, 11).

The aging process is associated with changes in lifestyle, socioeconomic factors, and biology, all of which contribute to the development of various diseases and injuries. As a result, the global healthcare system is experiencing an increasing burden in managing these emerging health challenges (12). Advanced age is recognized as a risk factor for vitamin D deficiency, a condition prevalent among the older adults population. Low serum vitamin D among the older adults is caused by various risk factors, including impaired skin synthesis, intestinal absorption, and hydroxylation in the liver and kidneys, and decreased dietary intake (12–14).

The association between vitamin D status and long length of hospital stay remains to be explored. The first aim of this study was to determine the potential association between serum level of 25(OH)D and extended length of hospital stay in geriatric patients. The second aim was to estimate the difference in hospitalization days between geriatric patients with different vitamin D statuses.

## Materials and methods

### Study population

The study sample comprised of 422 patients admitted to the geriatric department of a hospital in Piekary Slaskie (Silesia region, South Poland) from 2013 to 2016. In total, 242 older adults inpatients who met inclusion criteria were recruited in the study. Marked physical and/or mental impairment, cancer, liver disorders, decompensated thyroid disease, use of anticonvulsants or glucocorticosteroids, and use of vitamin D supplements within 3 months before admission to the hospital excluded patients from the study. The study was conducted in accordance with the ethical standards (Declaration of Helsinki) and was approved by the Bioethics Committee of the Medical University of Silesia, Katowice, Poland. Participants provided informed consent before participating in the study.

### Methods

Blood samples were collected in the morning on the day of admission, samples were centrifuged to separate serum and the blood samples were not stored. The measurement of serum 25(OH)D was standardized.

Patients were divided into two groups according to the diagnostic threshold defining serum 25(OH)D concentration approved for Central Europe. The first group (deficiency group) consists of patients with vitamin D deficiency (*N* = 193)—serum 25(OH)D concentration equal to or below 49.92 nmol/L. The second group (suboptimal group) contains patients with suboptimal vitamin D status (*N* = 46)—serum 25(OH)D concentration between >49.92 and 74.88 nmol/L and patients with adequate vitamin D status (*N* = 3)—serum 25(OH)D concentration ≥74.88 and below 124.8 nmol/L (15).

Based on the length of hospitalization, patients were divided into two groups: the first group, named shorter hospitalization, consisted of patients with a length of hospital stay of up to 11 days. The second group, named longer hospitalization, comprised patients with a length of stay in the hospital of 12 days and above.

### Laboratory measurements

To measure the serum level of 25(OH)D (nmol/L) in the blood using ARCHITECT 25-OH vitamin D test (detection range: 9.984–399.36 nmol/L, % CV: ≤10), an enzyme-linked immunosorbent assay (ELISA). Each step of the diagnostic process was constantly monitored and controlled in the laboratory. Measurement was conducted according to a quality management system compatible with the standard EN-PN 9001:2008.

### Statistical analysis

The data were analyzed using the statistical software STATISTICA 10 PL (Tulsa, Oklahoma, USA). The Shapiro–Wilk test was used to evaluate the normality of the distribution of the analyzed variables. Normally distributed data were compared using Student's *t*-test or analysis of variance (ANOVA). Non-parametric data were compared using the Mann–Whitney *U* test and the Kruskal–Wallis test, respectively. Analyzed variables were expressed as mean ± SD (for normally distributed data) and as median, quartile lower, and quartile upper (for non-parametric data). A probability level of *P* ≤ 0.05 was considered to be significant.

## Results

### Characteristics of the study group

In total, 242 Caucasian patients, primarily women (172 women, 70 men), were enrolled in the study. The baseline clinical and laboratory characteristics of the study group are shown in Table 1.

**Table 1:** Baseline clinical and laboratory characteristics.

**Parameters**	***N***	**Mean (Q1-Q3)**	***N***	**Mean (Q1-Q3)**	***N***	**Mean (Q1-Q3)**	*P*-value^*^
	**Total group**		**Deficiency group**		**Suboptimal group**		
Age [years]	242	78.00 (72.0–83.0)	193	79.00 (74.00–83.00)	49	75.00 (67.00–80.00)	0.002
Length of hospitalization [days]	242	10.00 (8.00–12.00)	192	10.00 (8.00–13.00)	49	9.00 (8.00–11.00)	0.044
Serum 25(OH)D concentrations [nmol/L]	242	33.95 (26.96–45.18)	193	31.17 (24.46–38.69)	49	57.41 (52.92–62.90)	0.000
Fasting glucose [mmol/L]	242	5.38 (2.83–16.43)	193	5.38 (5.00–6.44)	49	5.22 (4.94–5.77)	0.089
Total cholesterol [mmol/l]	240	4.84 (4.12–5.80)	191	4.82 (4.07–5.83)	49	5.13 (4.43–5.65)	0.429
HDL cholesterol [mmol/l]	240	1.45 (1.14–1.74)	191	1.45 (1.14–1.76)	49	1.35 (1.11–1.66)	0.227
LDL cholesterol [mmol/l]	240	2.83 (2.10–3.61)	191	2.75 (2.03–3.61)	49	2.93 (2.41–3.63)	0.145
Triglycerides [mmol/l]	239	1.20 (0.94–1.58)	190	1.16 (0.94–1.57)	49	1.30 (1.01–1.58)	0.389
CRP [mg/l]	238	1.80 (0.70–4.20)	190	1.85 (0.70–4.10)	48	1.65 (0.50–5.25)	0.719
Aspartate aminotransferase AST [U/l]	242	17.00 (15.00–21.00)	193	17.00 (14.00–21.00)	49	19.00 (15.00–23.00)	0.164
Alanine aminotransferase ALT [U/l]	242	15.00 (11.00–20.00)	193	15.00 (11.00–19.00)	49	16.00 (12.00–22.00)	0.241
Gamma-glutamyltransferase GGTP [U/l]	237	20.00 (15.00–34.00)	188	21.50 (15.00–34.00)	49	19.00 (15.00–28.00)	0.669
Leucocytes (103/mm3)	241	6.1 (5.1–7.4)	192	6.1 (5.1–7.6)	49	6.1 (5.1–7.1)	0.673
Erythrocytes (106/mm3)	242	4.24 (3.95–4.57)	193	4.26 (3.93–4.57)	49	4.34 (4.06–4.59)	0.394
Hemoglobin (g/dL)	242	12.6 (11.7–13.4)	193	12.6 (11.8–13.4)	49	12.7 (11.6–13.5)	0.680
Hematocrit (%)	242	38.8 (36.2–41.2)	193	38.5 (36.1–41.2)	49	39.7 (37.4–41.2)	0.299
Platelets (103/mm3)	242	217 (176–258)	193	219 (175–253)	49	214 (176–269)	0.863
Albumin (g/L)	130	35.0 (32–37)	111	35 (32–37)	30	63.9 (59–66.9)	0.066
Total protein (g/L)	118	64.0 (60–67)	88	64 (60.2–67)	19	37 (33–40)	0.975
TSH [uIU/ml]	233	1.37 (0.83–1.94)	187	1.37 (0.83–1.96)	46	1.35 (0.83–1.75)	0.756
Body weight [kg]	242	68.80 (59.60–78.60)	193	68.70 (59.30–78.10)	49	69.40 (60.20–82.90)	0.566
Height [cm]	242	156.50 (151.0–163.0)	193	155.00 (150.50–163.00)	49	160.00 (153.00–165.00)	0.101
BMI index [kg/m^2^]	242	27.35 (24.50–31.50)	193	27.30 (24.50–31.60)	49	27.60 (24.80–30.00)	0.944
Body fat [%]	242	34.90 (26.60–40.60)	193	35.00 (26.50–40.60)	49	34.60 26.70–40.50)	0.917
Muscle mass [kg]	242	41.10 (37.10–48.80)	193	41.30 (37.00–48.50)	49	40.80 (37.20–50.40)	0.801
Lean body mass [kg]	242	43.40 (39.10–51.40)	193	43.50 (39.00–51.10)	49	43.00 (39.20–53.10)	0.827

* Between deficiency and suboptimal, adequate group.

Patients with vitamin D deficiency had a longer length of hospitalization than those with vitamin D levels below 49.92 nmol/L−10.0 (8.00–13.00) days vs. 9.00 (8.00–11.00) days, P = 0.044.

Table 2 shows a comparison of health status between patients with deficient, suboptimal (and adequate) vitamin D levels. The frequency of comorbidities was not different between the analyzed groups, except for diabetes mellitus and arteriosclerosis, which were more common in the deficiency group.

**Table 2:** Health status/comorbidities between patients with deficiency and suboptimal (and adequate) vitamin D levels.

	**Deficiency group**	**Suboptimal group**	***P*-value**
Arterial hypertension *N* (%)	157 (81.3)	41 (83.7)	0.706
Diabetes mellitus *N* (%)	75 (38.9)	10 (20.4)	0.016
Hypercholesterolemia *N* (%)	52 (26.9)	18 (36.7)	0.177
Coronary artery disease *N* (%)	71 (36.8)	16 (32.7)	0.590
Heart failure *N* (%)	60 (31.1)	12 (24.5)	0.794
Arteriosclerosis *N* (%)	94 (48.7)	12 (24.5)	0.002
Alzheimer's disease *N* (%)	20 (10.4)	7 (14.3)	0.436
Anemia *N* (%)	52 (26.9)	13 (26.5)	0.954
Chronic kidney disease *N* (%)	34 (17.61)	7 (14.3)	0.579
Chronic obstructive pulmonary disease *N* (%)	17 (8.8)	4 (8.2)	0.886
Diverticulosis *N* (%)	48 (24.9)	9 (18.4)	0.338
Prior stroke *N* (%)	19 (9.8)	4 (8.2)	0.720
Hypothyroidism *N* (%)	27 (14.0)	5 (10.2)	0.485
Osteoporosis *N* (%)	20 (10.4)	2 (4.1)	0.172
Obesity *N* (%)	66 (34.2)	13 (26.5)	0.753
Overweight *N* (%)	71 (36.8)	21 (42.9)	0.753
Smoking *N* (%)	17 (8.8)	1 (2.00)	0.107

**Figure 1:**
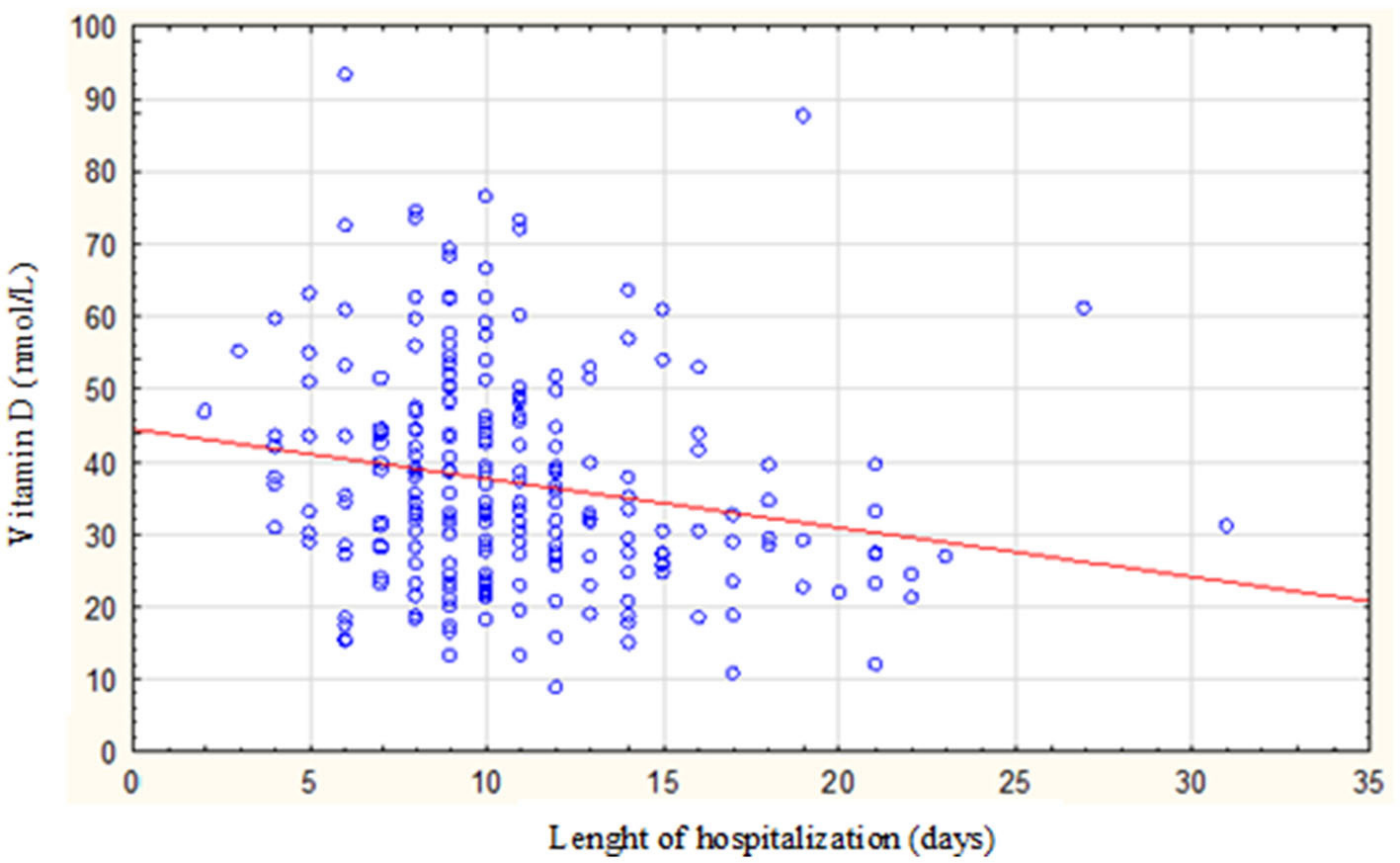
Correlation between the length of hospitalization and vitamin D status among the study population.

The length of hospitalization (in days) was found to have a negative correlation with vitamin D blood status (nmol/L) (*P* = 0.0005; *R* = −0.2243) (Figure 1; Table 3).

**Table 3:** The correlation between the length of hospitalization and vitamin D status among the study population.

**Vitamin D status in:**		***R***	***P*-value**
Total group	Length of hospitalization (days)	−0.2243	0.0005
Women		−0.227	< 0.0001
Men		−0.1800	0.1388

**Figure 2:**
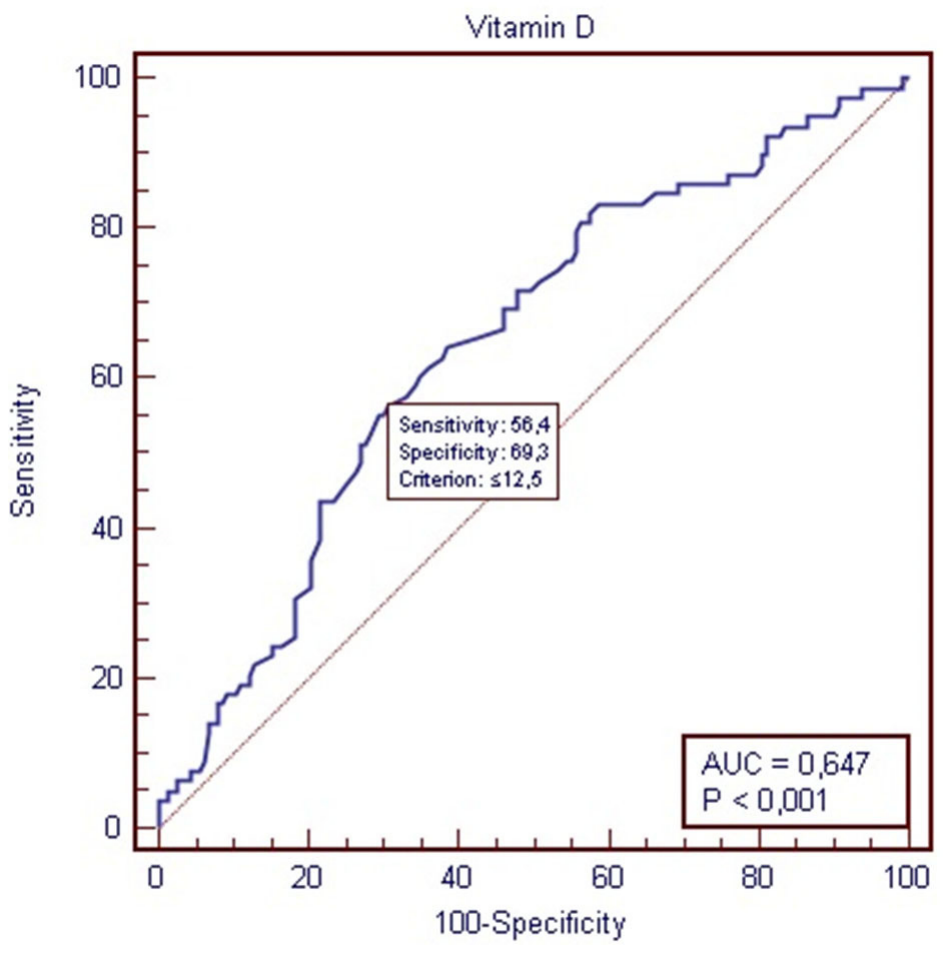
Receiver-operating characteristic (ROC) for vitamin D status and length of hospitalization.

Based on the ROC analysis (Figure 2), patients with a vitamin D concentration below 12.5 ng/ml (31.2 nmol/L) have a 47% higher chance of extended hospitalization (12 days or more). Patients with a vitamin D concentration above 12.5 (31.2 nmol/L) have a 77% higher chance of not experiencing extended hospitalization [AUC 0.674 (0.583–0.707) *P* = 0.0001; sensitivity, 56%; specificity, 69%; positive predictive value, 47%; negative predictive value, 77%].

**Figure 3:**
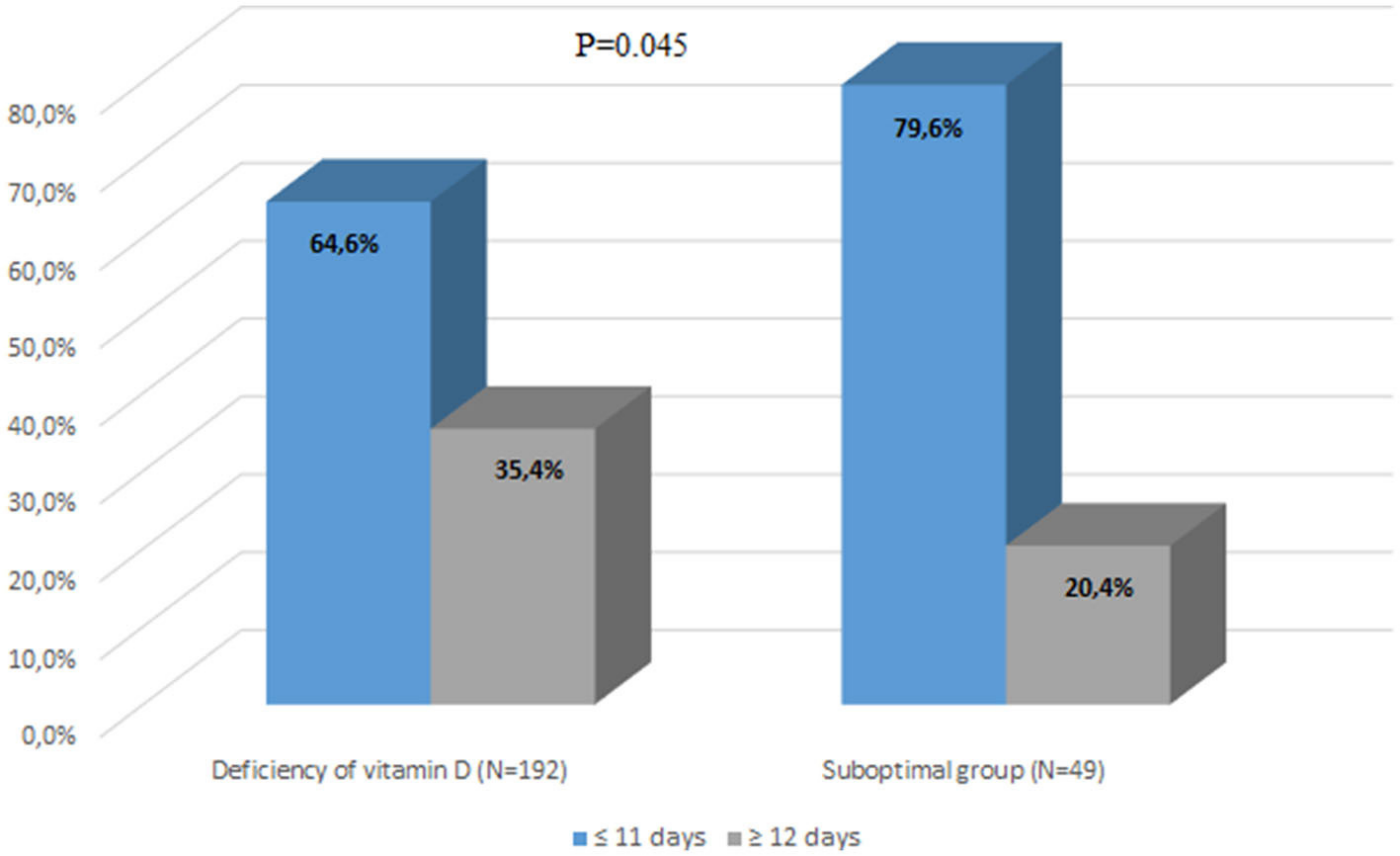
Comparison of length of hospitalization between the group with vitamin D deficiency and the group with suboptimal vitamin D status.

Most of the patients with suboptimal levels of 25(OH)D had a shorter length of hospitalization (≤11 days) than those with vitamin D deficiency (64.6%), *P* = 0.045 (Figure 3).

## Discussion

A prolonged hospital length of stay (LOS) has negative effects on patients increasing sarcopenia and a higher risk of hospital-acquired infections (16). Identifying demographic and clinical factors that contribute to prolonged LOS and creating a straightforward evidence-based predictive equation would be beneficial for healthcare planning and facilitating discharge planning. Some recently published studies have shown a connection between vitamin D deficiency and an increased risk of severe morbidity burden and low functional ability among older adults patients hospitalized in geriatric acute care units (11). Authors link vitamin D deficiency to severe morbidity burden and low functional ability in older adults patients and all-cause mortality in chronic heart failure in middle-aged and older adults (17, 18). Some of them show that lower vitamin D status is associated with greater severity of chronic diseases (regardless of the number of diseases) and a higher risk of clinical instability and mortality (7). Our study was conducted among older adults people admitted to the hospital with various causes. The relationship between vitamin D and a longer hospital stay was observed in our group of patients, similar to other studies that included older patients with specific medical conditions (9–11, 19–21). According to Braun et al. (6) deficiency of 25(OH)D prior to hospital admission is a significant predictor of short- and long-term all-cause patient mortality in a critically ill patient population.

As widely known, vitamin D has many benefits to the human body, especially among the older adults. Higher vitamin D levels are linked to a decreased risk of bone fractures, falls, autoimmune diseases, type 2 diabetes, cardiovascular diseases, and cancers. On the other hand, vitamin D deficiency is widespread among the older adults, and it should be considered a public health problem. In our study, the majority of the analyzed patients (79.75%) were found to have vitamin D deficiency, which was defined as having a serum 25(OH)D concentration equal to or below 49.92 nmol/L, whereas normal concentration is ≥74.88 and below 124.8 nmol/L. Only a small proportion of patients (1.14%) had adequate levels of vitamin D. The study also revealed that patients with vitamin D deficiency had a significantly longer length of hospitalization than those with vitamin D levels below 49.92 nmol/L. Furthermore, the research found a negative correlation between the length of hospitalization (measured in days) and vitamin D blood status (measured in nmol/L), indicating that as vitamin D levels decreased, the length of hospital stay tended to increase. The statistical analysis showed a significant relationship between vitamin D status and hospitalization duration (*P* = 0.0005, *R* = −0.2243). Finally, it was observed that patients with a vitamin D concentration above 12.5 nmol/L had a 77% higher chance of not experiencing extended hospitalization, suggesting that maintaining adequate levels of vitamin D may potentially reduce hospitalization duration. The authors acknowledge that based on the study of this small group of patients drawing any conclusions is not convincing. Additionally, they recognize that various factors (medical and non-medical) may influence the duration of hospitalization in this group. The analysis did not include the cause of hospitalization and intensity of comorbidity. However, the few conducted studies and literature analyses so far have revealed similar findings and results.

In 2013, three studies were published by Beauchet et al. focusing on the correlation between the concentration of vitamin D and the length of hospitalization in a geriatric acute care unit. First, Beauchet et al. showed a longer length of stay (LOS) in patients with hypovitaminosis D than individuals with adequate concentrations of vitamin D (respectively, 15.2 ± 8.2 vs. 12.1 ± 7.0 days, *p* = 0.017), indicating a mean difference of 3.1 days. Furthermore, this study was the first to demonstrate an inverse correlation between 25(OH)D concentration and LOS (*p* = 0.028; *r* = −0.14) (9). The following study found that patients with the highest LOS (≥14 days) had a higher prevalence of serum 25(OH)D deficiency (58.3 vs. 38.7%, *P* = 0.001) and male sex (58.3 vs. 28.5%, *P* < 0.001) than those with lower LOS (<14 days). Furthermore, serum 25(OH)D deficiency [odds ratio (OR) = 2.22, *P* = 0.001 for the unadjusted model; OR = 1.87, *P* = 0.012 for the fully adjusted model] and male sex (OR = 2.87, *P* < 0.001 for the unadjusted model; OR = 2.64, *P* = 0.001 for the full model) were found to be associated with a high LOS. The authors suggest that utilizing these risk factors to identify inpatients at risk of a long LOS may be beneficial in adjusting early care plans, leading to improved patient health and reduced hospital stays. Similar results were observed in our study (11). Beauchet et al. showed that vitamin D deficiency, delirium upon admission to the geriatric acute care unit, and male sex were all identified as individual risk factors for longer length of stay (LOS). Moreover, when these factors were combined, the risk of longer LOS significantly increased, resulting in a 4.8-fold higher likelihood of experiencing extended hospitalization (10). Based on the results of these studies, the authors suggest that vitamin D may serve as an easily accessible and cost-effective risk factor, and it can be integrated into clinical practice to identify inpatients at risk of longer hospital stays. By utilizing vitamin D status to adjust early care plans, healthcare providers have the potential to enhance patient health, shorten hospital stays, and reduce healthcare expenses (9–11).

Similar studies were conducted on patients hospitalized in other hospital departments due to different issues (5, 7, 8, 19–21).

The association between vitamin D status on admission day and length of stay was also observed in orthopedic patients scheduled to undergo elective hip or knee arthroplasty. Patients with vitamin D below 20 ng/mL had longer length of stay in the hospital (15.6 ± 7.2 days) than the group with normal serum 25(OH)D concentration (11.3 ± 7.9 days), *p* = 0.014. The results of the study indicate an inverse correlation between serum 25(OH)D level and the length of hospital stay in the orthopedic department when compared to patients with normal vitamin D levels, based on univariate analyses (*r* = −0.16; *P* = 0.008). Furthermore, in the multivariate analysis, after accounting for age, sex, primary musculoskeletal diagnosis, BMI, and comorbidities, low 25(OH)D levels remained significantly associated with a longer hospital stay (*P* = 0.002). Patients with vitamin D levels in the target range experienced a hospital stay that was 4.3 days shorter than patients with hypovitaminosis D. This suggests that low vitamin D levels may contribute to prolonged hospitalization even after considering these confounding factors (8).

In a study by Blay et al. conducted among patients admitted to the burn service for the initial management of burn injuries, interesting results were shown. Despite having similar baseline characteristics and severity of burn injury, patients with vitamin D deficiency or insufficiency experienced higher complication rates and longer stays in the intensive care unit and hospital than those with normal vitamin D levels (5).

Boccardi et al. (7) suggested that vitamin D status can be used as a biomarker for the burden of comorbidities and may indirectly impact clinical outcomes, including the length of hospital stay (LOS), among the older adults admitted to an acute care geriatric unit. Additionally, low vitamin D levels in nursing home residents (aged 81.5 ± 10.9 years) admitted to a community hospital were directly correlated with an extended hospital length of stay (13.72 ± 10.8 vs. 7.72 ± 4.1 days; *p* = 0.002) (19). Among surgical patients in critical condition, the same results were observed by Alizadeh et al. (20). Individuals with serum vitamin D levels below 30 ng/ml experienced longer stays than those with levels at or above 30 ng/ml (7.8 ± 5.1 vs. 4.05 ± 2.12 days, *P* = 0.003). Although vitamin D deficiency was linked to higher hospital mortality (25% in the deficient group vs. 22.2% in the sufficient group), it was not significant. This may be due to the positive impact of vitamin D on immune functions, reducing tissue dysfunction and mitigating the risk of organ failure and complications.

Matthews et al. (21) reported similar findings in patients admitted to the Surgical Intensive Care Unit. The severe vitamin D-deficient group had an average stay of 13.33 ± 19.5 days compared with 7.29 ± 15.3 days for the moderate deficiency group and 5.17 ± 6.5 days for the mild deficiency group (*P* < 0.002). Furthermore, the severe vitamin D-deficient group exhibited a mortality rate of 12.3% (17 cases), whereas, the moderate group had a rate of 11.5% (11 cases) (*P* < 0.125).

Finally, the authors highlight that it is essential to underscore that this study does not encompass data regarding vitamin D levels and outdoor time. Our previous research (22) revealed the absence of seasonal variations in vitamin D concentrations within this specific patient group. Notably, in Poland, even during the summer months, it is relatively difficult for an older adults person to produce an adequate amount of vitamin D through the skin. A reduced cutaneous synthesis of vitamin D is commonly observed in older adults individuals. Aging is linked to a notable decline in the ability of the skin to produce 7-dehydrocholesterol. Consequently, a 70-year-old person generates 75% less vitamin D through skin synthesis when compared to a 20-year-old person. This leads to lower vitamin D concentrations in the older adults, which persist year-round, regardless of the season, in comparison with younger individuals (22).

## Conclusion

In conclusion, the study findings indicate that lower serum levels of 25(OH)D in hospitalized patients within the geriatric department are linked to extended hospital stays. Vitamin D holds potential as a predictor of hospitalization duration in geriatric patients. Nonetheless, further research is imperative to account for additional factors affecting health status and hospitalization duration in older adults individuals.

It is difficult to establish a direct causal relationship between vitamin D levels and the length of hospitalization due to the potential influence of other variables. These variables include functional status score, illness severity, cognitive score, poor nutrition, comorbidity score, specific diagnosis or presenting illness, polypharmacy, age, and sex.

Maintaining adequate vitamin D levels may play a crucial role in promoting better health outcomes and functional independence among this vulnerable population.

## Study limitations

This study exhibited several limitations worth addressing. First and foremost, the absence of factors such as the cause of hospitalization and the severity of comorbidities presents a notable limitation. Additionally, the relatively small sample size and the imbalance between the sex distribution within the groups, as well as between the vitamin D deficiency and suboptimal groups, should be acknowledged. Specifically, it is important to highlight that only three patients exhibited adequate levels of vitamin D. Moreover, further studies would include frailty evaluation, including the assessment of movement ability.

## Data availability statement

The raw data supporting the conclusions of this article will be made available by the authors, without undue reservation.

## Ethics statement

The studies involving humans were approved by Medical University of Silesia KNW/0022/KB1/53/14. The studies were conducted in accordance with the local legislation and institutional requirements. The participants provided their written informed consent to participate in this study.

## Author contributions

JN: Conceptualization, Data curation, Formal analysis, Funding acquisition, Investigation, Methodology, Project administration, Resources, Software, Supervision, Validation, Visualization, Writing–original draft, Writing–review and editing. MJ: Data curation, Investigation, Visualization, Writing–review and editing. PJ: Data curation, Formal analysis, Software, Validation, Writing–review and editing. BH: Writing–review and editing. KB: Data curation, Writing–review and editing. JB: Software, Visualization, Writing–review and editing. BZ-S: Conceptualization, Resources, Writing–review and editing.
